# Supplementary material for: Clinical and prognostic relevance of sST2 in adults with dengue-associated cardiac impairment and severe dengue
Source: PLoS Negl Trop Dis. 2022 Oct 13;16(10):e0010864. doi: 10.1371/journal.pntd.0010864 (PMC9595566; doi:10.1371/journal.pntd.0010864)
Supplement: Attachment — Submitted filename: Responsetoreviewers_110822.docx [file pntd.0010864.s001.docx]

We appreciate comments from the reviewers and have edited accordingly to improve the manuscript for consideration.

Reviewer #1:

The objectives could be more clearly stated
The study design is appropriate
The population though appropriate, could be more clearly described
The sample size is underpowered within certain subgroups
Statistical analysis are appropriate, though additional methods could still be explored
Ethical and regulatory requirements were met

Summary: In this study the authors posit that “sST2 - soluble suppressor of tumorigenicity” was increased in adults with dengue in proportion with disease severity in the febrile and critical phases that may persist despite clinical recovery. Importantly, sST2 was a predictor of severe dengue in the critical phase, and elevated sST2 was also associated with dengue-associated cardiac dysfunction in the febrile, critical and early recovery phases.

Abstract: Line 3-4: Is the objective of the study to evaluate the utility of soluble suppressor of tumorigenicity (sST2) as a marker of severe dengue? Or is it to evaluate the utility of soluble suppressor of tumorigenicity (sST2) as a marker of cardiac impairment in severe dengue?
The objectives of this study are: 1) to explore the association of sST2 on dengue-associated cardiac impairment (a criteria for severe dengue); 2) to determine whether sST2 is a useful biomarker for severe dengue. We have edited the text to better reflect the objectives – line 15-18

Main body – Introduction
Line 15-18; 40-41; 225-226; 251-252: Regarding reference to the “lack of” prognostic biomarkers of severe dengue … is there any published evidence of the many predictive biomarkers of severe dengue? Maybe could summarize the historical evidence thus far in the background literature and mention evidence which certainly exists for children and youth (eg., Hb SC genotype, change in hemoglobin concentration, hepatic transaminitis with elevated LDH and CPK, multiple organ system involvement (etc), which were statistically significant predictors for severe dengue with hemorrhage and attributable mortality (viz: Lue, Frontiers in Medicine, 2022; Rankine-Mullings et al, Ebiomedicine, 2015; Elenga et al, J Infect Public Health, 2020; Kularatman et al, BMC Pediatrics, 2019; Sani, BMC Infectious Diseases, 2017; Prasad, PIDJ, 2020) and then say … However, prognostic biomarkers of cardiac malfunction as predictors of severe dengue need to be determined. In this study we sought to …
We thank the reviewer for this suggestion and have updated the text to reflect sickle cell diseases, change in haematological parameters and elevated enzymes as significant biomarkers of severe dengue Line 66-68. We acknowledged previously explored biomarkers; however, these biomarkers are not routinely used due to lack of specificity or sensitivity. We included in line 247, “lack of reliable biomarker” and explain the purpose of studying sST2 as a possible biomarker.

Background: What was the prevalence of dengue in the community when and where the study was being performed?
We have included the statement “…dengue is endemic in Singapore and the population experience periodic waves dengue.” Line 92

58-61 – Dengue shock syndrome (DSS). Have the terminology and criteria been since revised (refs # 3-7 now updated to WHO’s ref #17)?
We thank the reviewer for his comment. The term as used in the manuscript was meant to refer to the complication of shock (dengue shock syndrome) as defined in the World Health Organization 2009 Dengue Case Classification and not the 1997 WHO definitions. We have edited the manuscript accordingly to make this clearer.

Results

Reviewer #1: The analysis presented matches the plan
The results are clear
The numbers in the tables could be clarified
The figures are impressive

Results: What was the dengue serotype of patients? What serotypes were circulating in the community? Was ZIKA circulating, also?
We thank the review for the questions. We have updated table 1 to reflect serotypes. “44% were positive for dengue virus (57.5% DENV2, 37.5% DENV3 and 2.5% each DENV1 and 4. Table 1”

In our cohort, we detected DENV-2 and a shift towards DENV-3. To our knowledge ZIKA was not circulating during recruitment.

Lines 11, 128, Table 1: How many were severe dengue with hemorrhage? Would they have worse cardiac prognostic indicators than those without hemorrhage? Was there a statistical difference? Or were numbers when further disaggregated too small for meaningful comparisons?
We thank the reviewer for the questions.

We only had 1 participant with clinically severe bleeding. A recent study from Thailand observed DHF to be a significant risk factor for cardiac dysfunction (PMID34507547). Our numbers are too small for meaningful comparison.

Line 132 – correction myocardial “infarction”
We thank the reviewer for identifying the typo, we have corrected line 144

Table 1: Some of the numbers (mean? median?) and figures in the brackets (###) are not clear.
We thank the reviewer for the comment, we have included (IQR) in table 1, and line 152 reads “data are presented in median (IQR)…”

Tables 1, 2; Lines 280-282: With only one with clinical heart failure? one admitted to ICU? No cases of myocarditis? Pericarditis? Echocardiography anomalies? Or deaths? What then is the “clinical correlation” with laboratory results obtained for significant cardiac compromise reported here?
We thank the reviewer for the comments. We have acknowledged in the discussion the limitations are the small number of severe cases, and the observation of transient cardiac impairment in the cohort did not correlate with clinically relevant cardiac impairment. Line 290-293.

However, while a definite cardiac syndrome or compromise was not documented in our study due to no patient in the study having cardiac specific symptoms, the use of the whole body bio-impedance system to determine cardiac parameters including stroke index, cardiac index and Granov Goor index demonstrated that there is cardiac dysfunction according to disease severity which has not been clearly demonstrated before. The information may be informative for clinicians managing adult dengue patients who may be at risk of fluid overload and pulmonary oedema if fluid replacement does not take the cardiac dysfunction

Figures 1,2,3: Trends look impressive
Thank you.

Given multiple potential confounders, since there is a control population, should logistic regression modeling be done to factor in whether other variables may have been affecting outcomes?
We appreciate the reviewer comments. We acknowledge that hypertension could be a potential confounder. Line 280. However, because of the small number, the study lack power to robustly evaluate this.

**Conclusions**
Reviewer #1: In this study the authors posit that “sST2 - soluble suppressor of tumorigenicity” was increased in adults with dengue in proportion with disease severity in the febrile and critical phases that may persist despite clinical recovery. Importantly, sST2 was a predictor of severe dengue in the critical phase, and elevated sST2 was also associated with dengue-associated cardiac dysfunction in the febrile, critical and early recovery phases.

In discussing immunological mechanisms for dengue pathogenesis, is there a role for mentioning antibody dependent immune enhancement (ADE), which has been linked to severe dengue, after subsequent infection with another heterogenous dengue serotype, dengue type 2 serotype disease occurring after ZIKA infection; Dengvaxia vaccine (Soo K-M, PLOS One, 2016; Pawitan, Acta Med Indones, 2011; Stettler et al, Science, 2016; Katzelnick et al, Science, 2020; George et al, Sci Rep 2017; Sanofi, 2019)? Are/were India and also Indonesia – where this study was performed, undergoing a significant dengue and/or ZIKA surge? Although controversial, should ADE be mentioned and referenced, also?
We agree with the reviewer on the importance of ADE mechanism in severe dengue. However, in the current study, we explored on sST2 in dengue-associated cardiac impairment and the relevance of it as a biomarker to predict severe dengue. There is currently limited evidence to suggest ADE’s contribution in poorer cardiac function. Therefore, we feel that a section to described ADE will not fit well into the overall aim of the manuscript. Also our current cohort, to our knowledge, did not experience concurrent ZIKA.

Limitations: Would say in tropical and subtropical developing countries where severe dengue occurs, is it likely that these investigations would be useful to evaluate patients during an epidemic (eg., in Indonesia), when health systems are likely to be overwhelmed and severe cases of hemorrhagic dengue with high attributable morbidity are being encountered? Would you say this is a “pilot study”, given very small numbers overall, small proportions of cases to controls, imbalanced co-morbidities between cases (SD) and controls, lack of clinical correlations, given single cases of admission to ICU and cardiac failure with no cases of myocarditis, pericarditis, or deaths reported?
We thank the reviewer for the comments. We acknowledged the small severe dengue numbers in our cohort. We agree that in larger populations including Indonesia, Vietnam and India is likely to have the required numbers for more robust analysis. The current study requires further validation.

Public health relevance could be better outlined/stressed.
We thank the review for this suggestion, we have included this in the discussion. “… approximately 19.1% of febrile dengue patients…. Overwhelm healthcare…” line 244

Reviewer #2: This manuscript by Teo and colleagues provides additional information on the potential utility of sST2 as a maker of dengue disease severity. This is a very relevant area of research in the dengue field, as there is an urgent need for reliable biomarkers to predict progression to severe disease.

The objectives of the study and the authors' hypotheses are clearly articulated.

The participants section of the Methods could benefit from additional details. What were the ages you intended to recruit? Did you have a specific timeframe for fever duration to be able to participate (e.g. 7 days or less)? Was the disease severity classification done at admission, or at the end of the disease?
We thank the reviewer of the suggestions and have included the following

“…, >16 years (with no upper limit to age),…” Line 88.

We did not use a specific time frame restriction fever duration as an exclusion criteria. However, all patients were enrolled with a fever history of <7 days and the median febrile days on enrolment was 4-5 days. Table1. Line 91

Disease severity classification was done at the end of disease or discharge. This is to account for patients who may initially be admitted with dengue fever or warning signs, that further develop severe dengue during the hospital admission. Line 97

It seems that 24/36 dengue without warning signs patients in this study were hospitalized, 2/3 seems a little high as hospitalization is not typically warranted for these patients. Were 24 patients hospitalized as dengue without warning signs? Did they have additional risk factors that granted hospitalization? Was the hospitalization later in the disease because the disease severity increased? You mention in the document that none of the patients had cardiac compromise, but it would also be beneficial to include information on the specific type of severe dengue for the 11 patients included in this group (shock vs bleeding vs organ compromise). Did any patient die?

We appreciate the reviewer for the insights. Overall, 24 patients were hospitalized without warning signs. Based on a local study (Pang J, et al, BMC Infect Dis 2014), increasing age and cardiovascular risk factors (hypertension, etc) were found to be risk factors for severe dengue and ICU admission. With this data, dengue patients over 50 years of age, co-morbidities, or poor social support without warning signs are regularly admitted based on hospital guidelines.

We also updated in the text to reflect the 11 SD cases. “Of the 11 SD participants, three had dengue shock with two requiring inotropes, one had clinically severe bleeding, three had severe dengue hepatitis and eight had other end-organ impairment. No participant experienced clinical myocarditis. There were also no mortalities in our study.” No patient died. Line 146

(The information included in Line 278 regarding enrollment should be included in the methods and results)
We thank the reviewer for this comment. We have included details on the number of severe dengue patients enrolled in the results section. Line 146. However, we still think the sentence in line 278 (now line 289) is still informative, as it acknowledges that although we observed that sST2 was predictive of severe disease in the critical phase, the number of severe dengue in our study was relatively small.

A figure explaining the recruitment and follow-up process might be helpful to better understand the study procedures, including if DENV PCR testing was used, if you had information on DENV serotypes, and how previous dengue status reported in Table 1 was assessed, would also be useful to better define your population.

We thank the reviewer for the suggestions, and we have updated accordingly.

Dengue PCR was done on enrolment samples. 44% were positive for dengue virus (57.5% DENV2, 37.5% DENV3 and 2.5% each DENV1 and 4. Table 1
Previous dengue status was assessed via a convenience method, i.e., via history taking, checking of medical records and dengue serology if timing of dengue serology was appropriate. Line 159

Results

Reviewer #2: The results match the analysis plan.
Line 131: Do you mean they had history of hypertension, or they actually had high blood pressure during clinical assessment?

We thank the reviewer for the question. Participants had medical history of hypertension, through questionnaire at enrolment.

Line 134: Consider to include the range of days post-onset of illness at enrolment
We thank the review for this comment. In our setting, participants visited primary care at disease onset. They only come to the hospital if symptoms persist. Table 1 “days of clinical symptoms” (line 161) reports on the days post-onset based on medical history taking.

Tables:
Please revise your tables' titles to make sure that they are complete. The tables should be stand-alone.
Table 1: How was previous dengue assessed?

We thank the review for the comments. Previous dengue status was assessed via a convenience method, i.e., via history taking, checking of medical records and dengue serology if timing of dengue serology was appropriate. Line 159

Table 1: For the days of clinical symptoms, do the presented days correspond to the duration of each phase in number of days? How did you define early and late recovery phase?

We thank the reviewer for the questions. Days of clinical symptoms refers number of days after first reported symptoms Line 161.

We have provided definition of early and recovery phases in the text “Early recovery phase was defined as post critical phase and up to day 14 whereas late recovery was post day 15” Line 91.
Table 2: Providing normal/expected values for indexes would be helpful
We thank the reviewer for this suggestion, in table 2 we provided read outs from controls to severe as baseline in our population. While there are several reported ranges, however, due to difference in population, we do not feel it is accurate to provide an expected/normal range. In here, we provided a range for GGI, line 113.

Conclusions

Reviewer #2: The discussion section can benefit from additional edits, avoiding too much repetition of the information already presented in the introduction and the results.
We thank the reviewer for this suggestion and have edited accordingly.

Line 268: It is not completely clear in the methods and results, what do you describe as "cardiac dysfunction". To get to this conclusion, a better description of what is the definition of cardiac dysfunction is needed. Additionally, it would be important to include if any adjustment by other variables that can explain cardiac dysfunction in these patients was made.
We acknowledged the reviewer comments. In this study, we used a whole body bioimpedance device to measure cardiac functions in dengue participants and compared readouts with controls. Our study provided evidence of poorer cardiac performance in dengue participants. We edited throughout the text to reflect that in our cohort, we observed transient cardiac impairment or poorer cardiac performance where appropriate, and highlighted on our small sample size that reduced the power to detect possible severe outcomes.

We also highlighted that hypertension is a known risk factor of cardiac failure, however, due to small sample size we did not adjust for this potential confounder.

Limitations of the study are clearly stated.

Please consider to include additional discussion on the logistical/technical/economic aspects of using sST2 in clinical settings, as most areas affected by dengue are developing countries (is this an easy test to do? requires expensive supplies/devices? it is expensive? results can be obtained quickly? etc)
The reviewer raised a relevant point. However, we are not able to comment on this without further evaluation on sST2 prognostic values.

Reviewer #3: 1) Abstract, line 26 – “p < .001 for all” does not appear to be in accord with Table 2.

We thank the reviewer for pointing this out, we have corrected to <0.01 - line 27

2) Abstract, lines 35-36 – Suggest changing conclusion to something like “While further research is needed to demonstrate its clinical utility, sST2 may be a useful prognostic biomarker of severe dengue.”

We thank the reviewer for the suggestion and have updated the conclusion – “While further research is needed to demonstrate its clinical utility, sST2 may be a useful prognostic biomarker of severe dengue.” Line36

3) Author summary, lines 47-49 – Consider changing “poorer cardiac functions” to “transiently lower measurements of cardiac function” and replacing “good prognostic biomarker” to “possible prognostic biomarker.”

We thank the reviewer for the suggestions and have updated – “… with lower measurements of cardiac performance…” Line 48

“…be a possible prognostic…” Line 49

4) Introduction, line 71 – Based on references 12 and 13, the words “proportionate to” should be replaced by “associated with” to be accurate and precise.

We thank the reviewer for pointing this out. We have updated the to read “…were associated with …” line 77

5) Materials and Methods, lines 82-83 – Please describe how the study defined early and late recovery phases.

We thank the reviewer for this suggestion. We have updated this “Early recovery phase was defined as post critical phase and up to day 14, and late recovery phase was post day 15.” Line 91

6) Materials and Methods, lines 90-91 – Did all three criteria for have to be fulfilled in defining the critical phase? How were these determinations made?
We thank the reviewer for the question

We defined the critical phase according to the day with the lowest platelet count, along with the highest hematocrit and defervescence pattern, Line 1005). If there were discrepancies, for e.g. if the platelet count was 21 on day 7 of illness and platelet count was 24 on day 6 of illness and patient had defervesced on day 6 of illness with a higher haematocrit on day 6, then day 6 of illness (instead of day 7) would be considered the critical phase. Discrepancies occurred for cases with platelet counts varying <5 × 10^3^/µL.

Results
Reviewer #3:

Table 2 present interesting data that describe several measures of cardiac function and cardiac biomarkers kinetics during the four illness phases and stratified by disease severity. These are complex data that show a dynamic cardiovascular physiology during the natural history of dengue. This manuscript would benefit from the authors’ viewpoint on why the cardiac index in dengue patients starts higher than controls during the febrile phase (though not statistically significant) and then proceeds to decrease during critical and early recovery phase before returning to values close to controls during late recovery. The same line of thinking could also be applied to changes in the troponin T and NT-proBNP levels. While several analyses of differences among the 4 groups are presented, an analysis of their ability to distinguish severe disease from DF or DWS (especially during the febrile phase) would help the reader evaluate their prognostic value.

We thank the reviewer for the suggestions. The actual mechanisms underlying our observations as pointed out is likely to be complex and cannot be addressed by our observational study. The increase in the cardiac index in the febrile phase (albeit not statistically significant) may be due to the increase in the heart rate in the dengue patients. This is consistent with stroke index that is significantly lower in the severe dengue vs controls in the febrile phase.

There are significant differences in Trop T and proBNP levels when comparing dengue subjects to controls, however, within the different disease groups, there are no significant differences. Line 223.

Two possible reasons for the observations -1) the smaller number of severe samples is likely to limit the power to analyse; 2) our group did not consist of subjects with severe dengue cardiac dysfunction, the transient response in here, suggests possible cardiac complications. However, findings need to be validated in a larger cohort.

The main message of Figure 3 is that sST2 correlated with several of the measures of cardiac function (although I would suggest showing all data, especially during the febrile phase, even if not significant. However, if these measures of cardiac function are not predictive of disease severity, then it does not reinforce the argument that sST2, as a surrogate marker for these measurements, would assist in predicting severity. If data are available of the clinical relevance of these cardiac parameters to the clinical picture, these should be included to justify that a surrogate marker would be of clinical utility.
We appreciate the comments from the reviewer. In the text, we provided the association of sST2 with cardiac parameters in febrile phase Line 227.

In Figure 3, we aim to provide a comprehensive figure to highlight on the major findings. In this study, we explored on the association of sST2 as a possible marker of dengue-associated cardiac impairment and we demonstrated sST2 correlated with several poorer cardiac functions in Fig 3. We acknowledged in our discussion that one of the limitations was the small number of severe dengue cases, and larger cohort is needed to validate or findings. Line 292-295

5) Results, line 131-132 and Table 1
Data presented in Table 1 do not clearly indicate that 67% of severe dengue cases were baseline hypertensive and the myocardial infarction percentage in the table does not match the text.

We thank the reviewer for pointing out. We have corrected the values Line 144

How was previous dengue infection determined as shown in Table 1?

We thank the reviewer for the questions. We have included in the text “Previous dengue status was assessed via a convenience method, i.e., via history taking, checking of medical records and dengue serology if timing of dengue serology was appropriate.” Line 159

Days of clinical symptoms – The Table description is unclear. Are the days shown the full range (not an interquartile range) for each illness phase. Presumably blood samples were taken during the days indicated.

We thank the reviewer for the questions. Data presented as median and (IQR) Days of symptoms refers to “days after first reported symptoms” Line 166.

Hospital outcomes – Please describe the 1 ICU admission relevant cardiac conditions, if any. Did the patient require inotropies/vasopressors and/or cardiac pacing?

We thank the reviewer for the questions and have included in the text “Of the 11 SD participants, three had dengue shock with two requiring inotropes, one had clinically severe bleeding, three had severe dengue hepatitis and eight had other end-organ impairment. No participant experienced clinical myocarditis. There were also no mortalities in our study” Line 146

6) Results, Table 2 – In the p value column, please indicate whether the comparison is all 4 groups or comparing controls to all dengue cases combined.

We thank the reviewer for the comment. We have updated the manuscript it now reads “ANOVA/Krauskal-Wallis for comparison across groups.

7) Figure 2 – Graphs A and B have the same Y axis scale (0-40) and C (0-80). Please standardize axes for easier comparison.

We thank the reviewer for the suggestion and have standardised the axes.

8) Results, line 171 – The sentence as written is unclear. The authors seem to be saying that for the 5 severe disease patients with febrile phase samples, the AUROC value was 0.74.

We thank the reviewer for this suggestion. We have updated it now reads “In the febrile phase, we only had five subjects with SD, and the AUROC on sST2 as a biomarker was…” Line 189

6) Results, lines 172-176 – What is being compared to give p value results for AUROCs is unclear.

We thank the reviewer for the question. The AUROC in out paper is a probability model that measures the ability of sST2 to distinguish between severe dengue and non-severe dengue. It plots the true positivity rate (Y axis) against the false positivity rate (X axis). The p value of <0.05 in our study suggest the AUROC model (AUROC 0,79) using sST2 as a marker, is able to predict severe dengue (plotting true positives vs false positives) compared to a AUROC of 0.5 (which by definition cannot distinguish severe dengue from non-severe dengue) in the critical phase at a statistically significant level.

7) Results, lines 177-183 - What was the timing of the sST2 elevation in relation to that of increased hematocrit and drop in platelet count?

We thank the review for this question, the observations in here are sST2 readouts in the critical phase. Line 203

8) Results, line 196 - Are you describing differences between controls to all dengue cases combined (comparison between 2 groups) or differences among all 4 groups? Table 2 appears to be comparing among all 4 group which should be precisely worded in these results.

We thank the reviewer for highlighting this. Kruskal-wallis analyse change across the groups. It now reads “… in the critical phase, SI, CI and GGI measurements were significantly lower in dengue patients, and we observed a trend of lower readouts as disease severity progress.” Line 219.

9) Results, line 209: Higher troponin had an inverse relationship to disease severity according to table 2 in the febrile phase. Also, nt-probnp was significantly different between the dengue (unclear if combined dengue or stratified by severity analysis) and controls in the febrile and critical phase, but did not appear to have any difference among dengue severity (an analysis that should be shown, even if not significant).

We thank the reviewer for the comments. The differences reported compare the differences across the four groups, thus K-wallis tests were performed. Line 222. When we compared within the group, eg DF vs DWS, we did not observed significances. Line 224

10) Results - The authors should make clear whether any of the severely ill patients developed shock and that none of their study patients died. Did any of the non-dengue controls have a severe illness?
We thank the reviewer for this suggestion. We have included this in the text Line 146
--------------------
Conclusions

Reviewer #3: The authors present a study of sST2 (soluble suppressor of tumorigenicity, an interleukin-1 receptor group member) as a biomarker for severe dengue in 90 NS1 antigen confirmed dengue adult patients with disease severity classified according to 2009 WHO criteria and 30 non-dengue adult controls in Singapore. Dengue patients had serial quantitative testing of sST2 in each of the febrile, critical, early recovery and late recovery disease clinical phases. The study also measured cardiac performance indices and biomarkers of cardiac impairment, Troponin T and NT-proBNP, and correlated these with sST2 levels as well as with maximum hematocrit increases indicative of vascular leakage, and platelet counts. The study observed statistically significantly higher sST2 levels in dengue patients compared to controls, and significantly higher sST2 levels in severe dengue patients compared to dengue fever and dengue warning sign patients during the critical stages (5-7 days post onset). Area under ROC curve analysis suggested sST2 levels could be a useful biomarker for severe disease, particularly in the critical stage.

This study is reasonably rigorous in its assessment of sST2 as a biomarker for severe dengue in adults, particularly in its use of serial measurements in different dengue clinical phases in the study subjects, and acknowledges the study limitation of only 11 severely ill patients including only 5 severe cases identified during the early febrile stage. The study adds to literature regarding sST2 in severe pediatric dengue illness and limited data in adults. A biomarker to identify risk for severe dengue illness early in the course of the disease would be useful as only a small percentage medically attended dengue patients develop severe illness and clinical decompensation can occur rapidly during the critical stage. While the study is useful, the authors also acknowledge in the discussion that further evaluation of sST2 would be needed before adopting routine testing for sST2 in dengue clinical management protocols. For example, the area under ROC curve values for sST2 in this study are not greater than that seen using simple clinical warning signs without lab testing to predict severe disease as studied in Cambodian children in reference 24.
The study is reasonably well written, although some editing as suggested by “editorial comments” below would be desirable.

I would caution the authors’ from using the term cardiac dysfunction (line 268 and 288) to describe the correlation to the noninvasive measurement of cardiac function since they, for the most part, stay above 2.5 which would be considered the normal range for the cardiac index. The clinical significance of these measurements could be strengthened if data were available showing that these low normal cardiac indices resulted in signs/symptoms of heart failure, such as lower extremity edema or pulmonary edema, or sequelae of low cardiac output state leading to tissue hypoperfusion like elevated lactic acid or cyanotic/cool extremities. Other cardiac outcomes such as need for inotropic/vasopressor support or major cardiac assist devices or death due to cardiovascular complications should be presented if these are available. Without these data, these values could be argued to represent normal cardiovascular physiology that is compensating for a dengue disease state.
We thank the reviewer for the comments. We acknowledged that in our cohort, various cardiac markers did not fall below the “normal” range. However, we observed poorer cardiac performance in our dengue cohort compared with controls. We updated to the manuscript to better reflect this “decreased in cardiac performance” and “transient cardiac impairment” as appropriate. We also included the description of our severe dengue cases (Line 146)

11) Discussion, lines 229-231 – Consider refining this statement. Your data show that sST2 was inversely correlated with some cardiac measurements at some time points in disease course, but not consistently. And also, not during the febrile phase when it might be most helpful in guiding treatment. Also, while it was correlated with some cardiac biomarkers, the cardiac biomarkers themselves were not necessarily correlated with cardiac measurements and, in turn, those cardiac measurements were not necessarily correlated with dengue severity.

We thank the reviewer for this suggestion and have highlighted in the discussion that this was observed in the critical phase. Line 253.

We provided the diagnosis of severe dengue in the current cohort Line 146

We also highlighted that one of the limitations is lack of severe cardiac impairment in our dengue cohort, the current observations suggest transient cardiac impairment also reported in other Asian cohorts (PMID 17428513, PMID 9579814). Nevertheless, our results suggest sST2 to be associated with poorer cardiac performance, however, further study is needed to validate findings.

12) Discussion, lines 234-236 – The authors should note that there was a trend towards higher sST2 levels with disease severity, but not necessarily statistical significance at all time points.

We appreciate the reviewer’s comment. Our data showed that in febrile, critical and early recovery phase, sST2 levels to be significantly elevated compared to controls, in particular, sST2 levels were the highest in the severe dengue group. We also provided new data on change in sST2 levels and within the dengue groups. Line 195

13) Discussions, lines 241-244 - Unless these data are placed in supplementary material, consider leaving this out, since the readers cannot easily evaluate these data for themselves. Additionally, if you will be including this statement, please place in the results.

We thank the review for the suggestions and have removed the section.

14) Discussion, lines 247-248 - In Figure 2, it appears that sST2 levels go down as the disease progresses for all 3 disease severity states. When you state that to “sST2 levels appeared to remain elevated in the early and late recovery phases, especially in the SD group” are you referring to a specific threshold by which they remain elevated?

We appreciate the reviewer’s comment. In here, Table 2 demonstrated higher sST2 levels in the febrile, critical and early recovery phase compared to controls. In Figure 2, in the SD cases, sST2 levels were higher in the febrile and critical phase (slight decrease) We have reworded to improve on clarity Line 266.

15) Discussion, lines 252-255 - If one is trying to argue that sST2 is important for predicting progression to SD, the best data are those that focus on comparing 3 dengue severity categories. However, sST2 was not significantly higher in severe dengue compared to dengue fever and dengue with warning signs in the febrile phase. This was only true in the critical and early recovery phase.

We thank the reviewer for this comment. We agree that to predict disease progression, sST2 levels should be determine in the febrile phase, in Fig 1B left top panel, there is a trend of increasing sST2 levels with increasing disease severity, however, we acknowledge that the small sample size (n=5) has underpowered the analysis. In contrast, in the critical phase where usually severe dengue manifest, we have 11 SD cases, and sST2 levels is statistically significant. The AUROC readouts suggest sST2 may be a good biomarker, and larger cohort is needed to validate our findings.

16) Discussion, line 258 - 0.79. But 0.78 in the results.

We thank the review for highlighting this. We have corrected our typo. AUROC read out is 0.79, line 191

17) Discussion, lines 267-270 - sST2 levels were only associated with cardiac measurements in the critical and early recovery phase. You reported this as not associated with some measurements in the febrile phase. Also, none of the data presented show cardiac measurements (either in SI or CI) that were in the pathologic range or that this could be considered cardiac dysfunction by a clinical measure. Consider rephrasing for as "transiently lower measurements of cardiac function in patients with dengue" as they were not clinically significant.

We thank the review for this comment and have edited the manuscript to clarify our discussion “…. sST2 levels to be implicated with transient decrease in cardiac performance…” Line 277

18) Discussion, line 277 - “utility as a biomarker of SD as compared to a single time point.” Perhaps one should say “possible biomarker of SD when taken at specific time points” since the authors did not analyze change in sST2 levels taken as serial measurements in the same patient as a prognostic factor for dengue.

We thank the reviewer for the suggestions. We included the change in ΔsST2 levels over time and in the diseased groups to better highlight the kinetics of sST2. Line 195. Observations demonstrate per unit drop in sST2 levels in each study visit, and within the disease group (dengue fever as comparator) per unit increase in sST2 levels in increasing severity.

19) Discussion, lines 285–286 -- Consider rewriting the conclusion given that sST2 was only statistically significantly increased by disease severity in the critical and early recovery phases.

We thank the reviewer for the suggestion. We have reworded accordingly. It now reads “… sST2 was increased in adults with dengue compared to controls, and levels were significantly elevated in proportion to disease severity in the critical phase…” Line 297

120) Line 288: See general comments for using “cardiac dysfunction.”
We updated to “transient cardiac impairment and decreased cardiac performance” accordingly.
--------------------

**Editorial and Data Presentation Modifications?**


Use this section for editorial suggestions as well as relatively minor modifications of existing data that would enhance clarity. If the only modifications needed are minor and/or editorial, you may wish to recommend “Minor Revision” or “Accept”.

Reviewer #1: Minor revision

Reviewer #2: Line 20: I think the total samples was 120, as listed below in line 127, but here you list 122. Please check. – Corrected - 120 samples
Line 16 and Line 40: Use "biomarkerS" instead of "biomarker" -updated
Line 22: NT-probBNP should be spelled out here - updated
Line 49: Not sure the last part of this sentence can be claimed with the authors' findings – Updated line 52
Line 52: Dengue is mostly asymptomatic, suggest to revise the language -updated
Line 55-64: Introduction section would benefit for a more detailed explanation of how severe dengue manifests to include severe bleeding and organ involvement, besides shock. – we thank the reviewer for this comment, in this study we investigated on dengue-associated cardiac performance, and the mechanism behind this is unclear. Therefore, we feel that to described on other causes of severe dengue would deviate from the main aim of this study. We provided several references that described on the pathways of severe dengue (REF 8-10)
Line 63: Please consider to use the word "critically'' more sparingly throughout your document – We appreciate the comment and have removed unnecessary usage of critically.

Line 63: Consider to include that many biomarkers have been studied and suggested as potential predictive biomarkers of severity in dengue – updated line 66
Line 69: Do you mean "in" instead of "and"? – updated

Line 86: Please check language, at it is written now it looks like you are saying that patients who had dengue in the previous 6 months were enrolled as controls. Is that what you mean? – updated line 96, “who did not have a dengue episode in the past 6 months...”
Line 93: You mention "each study visit" but it is not clear from the methods description how this visits occur? Does this mean not all patients were hospitalized? Or "visit" refer to each encounter with the researchers? – We have clarified line 104. Parameters measured at febrile, critical and recovery phases (followed up).

Line 101: Please consider to add an explanation of hoy you are using a cut off of <10 or EF<55%, for those not familiar with what this means – updated for Line 113
Line 170: I cant find figure 2D in the document, is this 2C? – updated on typo. Line 188, indeed 2C
Line 227: Lower age range seems to be 29 by table 1, not sure your population corresponds to older adults – we thank the reviewer for this comment. We updated to in our cohort of adults Line 254
Line 253: Do you mean significantly higher when compared to the other dengue clinical classifications? – There is a trend of higher sST2 levels in SD group in febrile phase and levels maintained in the critical phase. We have updated accordingly, line 267

Reviewer #3: Lines 20–21: Consider “plasma samples collected from 36 patients with dengue fever, 43 patients…” -Updated Line 20
Line 34: “sST2 levels were raised in...” Consider changing to “were elevated in patients with dengue.”- updated line 35
Line 42: “valuable biomarker in patients…” consider specifying outcomes for which it was predictive (e.g. mortality, readmission, etc.). – updated, we have removed the sentence and replaced with “…further research is needed to demonstrate its clinical utility, sST2 may be a useful prognostic biomarker of severe dengue” Line 36
Line 53–54: Although severe dengue can occur with any case of dengue, references to WHO guidelines on early recognition of warning signs and low mortality with appropriate treatment should be referenced. – we updated the references line 61

Lines 59–64: These sentences should be revised to focus on either dengue shock syndrome, severe dengue, or explain the relationship of dengue shock syndrome to severe dengue when switching terms. – we updated the text to provide clarity, Line 59 described possible symptoms of severe dengue – vascular leakage, that may then progress to other outcomes including shock. These symptoms are considered severe dengue. Line 59

Lines 171–172: Is this discriminating SD from DF and DWS combined? Please explicitly describe what

sST2 is discriminating between. –

We thank the reviewer for the question, we have included in the text looking at severe dengue cases or DWS and SD group. Line 192

Line 175: “DWS and SD patients” – Please put an n if you put one for all other AUROC analyses. –

we have updated n =62 line194

Lines 199–201: “In the current study, there were significant differences in troponin-T and NT-proBNP levels in the febrile and critical phases between dengue patients and controls, but not among the SD, DWS and DF groups” - This test statistic is not shown in the table. If reported as not significant here, it's helpful to still report the p-value in the text. –

We thank the reviewer for the suggestion; however, we did not include the P value between the dengue groups, firstly not to confuse readers and also we feel that these values do not add value to the current manuscript. Thus we only provided significant readings

Lines 260–263: Interesting finding from that paper, but the data presented in this paper did not look at change in sST2 levels, so it’s difficult to make that comparison. Consider removing.

We thank the reviewer for the comment. We included the change in ΔsST2 levels over time and in the diseased groups to better highlight the kinetics of sST2. Line 195. Observations demonstrate per unit drop in sST2 levels in each study visit, and within the disease group (dengue fever as comparator) per unit increase in sST2 levels in increasing severity.

Lines 280–282: Consider adding the limitation that there are no clinical cardiac data that are correlated with the measurements of cardiac output. This is a larger limitation than simply not having myocarditis cases. – updated Line 301

Editorial comments
1) Abstract, line 24 – Suggest “… during the different phases for dengue subjects…” – updated line 24
2) Abstract, line 26 – sST2 typo error as sS2 – corrected line 27
3) Author summary, line 40, and Discussion line 225 – Suggest delete “critically,” a word consistently overused in the manuscript, “The lack of a reliable biomarker…” – We have edited the manuscript accordingly line 42 and line 248
4) Author summary, line 43 – Suggest “…we investigated the potential utility of sST2…” – updated line45
5) Author summary, line 48 – Suggest “.. this work provides novel evidence on the potential utility…- updated line 50
6) Introduction, line 57 – Suggest shorten to “…features of SD, …, is vascular leakage that may lead to potential fatal cardiovascular collapse…” – updated line 62
7) Line 58: Consider “cardiovascular collapse” should be “circulatory compromise” or “circulatory collapse” – updated line 60
8) Introduction, line 63 – Suggest delete “Critically,” – deleted line 72
9) Line 128: “recruited, and their plasma were…” Consider breaking into two samples and changing language to “Their plasma samples were assayed…”- updated line 140
10) Results, line 131 – “67%” belons after “hypertensive”- updated line 143
11) Results, line 132 – “infarction” missplelled – corrected line 144
12 Results, line 146 – Suggest delete “Critically,”- removed line 159
13) Results, line 170 – Appears that Fig 2D should be Fig 2C. – corrected line 188
14) Results, lines 196-7 – Suggest “…GGi measurements were significantly lower in severe 18dengue patients only in the critical phase.” – updated line 219
15) Results, line 203 – Typo, should be “…there were...” – updated line 226
16) Discussion, line 226 – Suggest delete “In here,” – removed Line 245
17) Discussion, line 245 – Appears that should read “…less severe in wild-type mice,…” updated “… sST2 knocked out mice… compared to wild type mice” – line 259
18) Discussion, line 251 – Should read “Biomarkers to predict SD are lacking…” – removed for better read line 266
19) Discussion, line 257 – Suggest replace “Critically” with “Importantly” – updated line 270
