# Supplementary material for: Clinical and prognostic relevance of sST2 in adults with dengue-associated cardiac impairment and severe dengue
Source: PLoS Negl Trop Dis. 2022 Oct 13;16(10):e0010864. doi: 10.1371/journal.pntd.0010864 (PMC9595566; doi:10.1371/journal.pntd.0010864)
Supplement: Attachment — Submitted filename: response to reviewers 200922.docx [file pntd.0010864.s002.docx]

Reviewer #1:
Summary: This pilot study has shown that sST2 levels were elevated in patients older than 18 years with dengue especially in cases of severe dengue and increased sST2 levels were associated with cardiac indicators suggesting lower cardiac performance. They conclude that while sST2 maybe a useful prognostic biomarker of severe dengue, further research is needed to demonstrate its clinical utility.
Assessment: The authors have made the relevant requested corrections. The revised manuscript is now suitable for publication, with minor and other edits, viz:

Lines 19-25 and lines 26-33: Suggest separating the methods (line 19-25) from the results/principal findings (lines 26 to 33), under separate subheadings and paragraphs, for clarity.

We thank the reviewer for this suggestion and have updated the manuscript

Line 20: Suggest adding patient age for clarity, viz: “… patients aged >16 years …”

We thank the reviewer for the suggestions and have updated. It now reads “Plasma samples, aged >16 years, collected…”

Line 75: Suggest including “dengue” in this sentence to make the definitive link between “severe dengue, mortality and sST2”. Reference (17-18)

We thank the reviewer for this comment. In this sentence, we highlighted sST2 as a biomarker for severity and mortality in heart failure (now REF 18,19). In the subsequent sentence, we then discussed sST2 as a marker in severe dengue (now REF 20-23).

Lines 76-77; line 255: How old were these “younger” patients? What was/were their age range/s? References (19-22)

We thank the reviewer for the comment, we have included the median age range line 77. It now reads “In younger dengue patients (median age range 3 – 25 years) ...”

Line 147: Would spell out the “8 with other end-organ impairment” and how many organ systems (eg., range, median) were involved per patient, for clarity. These could have adversely contributed to the outcomes.

We thank the reviewer for this suggestion and have updated. Line 150 “kidney injury and hemophagocytic lymphohistiocytosis”

Line 246: Suggest removing “but”

We thank the reviewer for the suggestion and have removed “but” – Line 248

Table 1, Results, Limitations: Given DENV endemicity in this region and the heterogenous serotypes that were co-circulating (44% positive for DENV: 57.5% DENV 2, 35.5% DENV 3 and 2.5% each for DENV 3 and DENV 4; with shift from DENV 2 to DENV 3) and dengue serotype heterogeneity with repeated infections (asymptomatic infections being the vast majority – not shown here) is a definitive risk factor for severe dengue, shouldn’t these results be “spelled out” in the narrative of the results and mentioned in the limitations (as a confounder), as this is a recognized factor risk factor for severe dengue?

We thank the reviewer for the comments. We agree that secondary dengue is a risk factor for severe dengue. Despite dengue being endemic in Southeast Asia, in Singapore, for reasons that are less clear, severe dengue only manifest in a very small portion of the population compared with Vietnam or Thailand. Also, in Singapore, the shift from serotype 2 and 3 is because of the periodic episodes of dengue outbreak, and surveillance on whether an induvial experienced two different strain of DENV infections is often not documented. We believe that good vector control may be one of the reasons on reduce risk of secondary dengue in Singapore.

In our cohort, secondary dengue was uncommon, and we did not have data of previous infected serotypes in individuals with secondary dengue. Therefore, to include secondary dengue as a confounder in here would overestimate a potential confounder that is less prevalent in this cohort. Nevertheless, we did highlight that one of the limitations is the lack of severe dengue cases or cases with more severe form of cardiac impairment.

Table 1, Results: Suggest adding a footnote that there were no recognized cases with co-morbidities of obesity, respiratory disease (including asthma), diabetes, renal failure, infectious diseases, as these are recognized risk factors for severe dengue and maybe confounders (like hypertension, already “spelled-out”) for some of the outcomes you have described.

We thank the reviewer for this suggestion, we have included a footnote. “No recognised cases of severe dengue with co-morbidities such as obesity and diabetes”

Thanks for the opportunity to review a second reiteration of your manuscript.
We thank you for your time

Reviewer #2: Line 142. Consider to use different language to refer to hypertension as a preexisting condition throughout the document, as this could be confusing to your readers. Suggest to use "history of hypertension" instead of being "hypertensive", the latter may seem that you are referring to the actual blood pressure measured during disease, not the report of an existing condition.

We thank the reviewer for the comment. We have updated this. Now reads “… had recorded history of hypertension…” Line 144 and line 287.

Line 145. Table 1. The distribution of severe dengue types add to more than 11, so I assume patients had more than one severe dengue manifestation. Consider reporting the n for those with a singular manifestation, and then using something like "XX patients had a combination of 2 or more severe dengue manifestations"

We thank the reviewer for this suggestion. We included the statement “Five patients had at least two complications”. Provide a breakdown of on the combination for everyone is likely to complicate the methods.

Line 149. As recommended before, your tables should be stand alone i.e., if I read the title I can tell who are your population, when and where. Please check the titles and complete them.

We thank the reviewer for the suggestion. We have updated the manuscript. It now reads “**Table 1:** Baseline Characteristics of controls, dengue fever, dengue with warning signs and severe dengue patients, and clinical outcomes of dengue participants” and “Table 2: Longitudinal comparison of cardiac parameters and plasma cardiac biomarkers in dengue fever, dengue with warning signs, severe dengue patients and controls”

Line 158. History taking and medical records can be very unreliable for determining dengue previous infection. As you did not find any difference by this variable, consider using only serology if timing was appropriate to determine who had previous dengue infection.

We thank the reviewer for the suggestion and have removed history taking… Line 162

Line 301. This is a very complex sentence. Consider to delete "dengue associated" or to rephrase
We are unsure on the reviewer’s suggestion.

Our results provide evidence of sST2 to as predictor of severe dengue in the critical phase, and elevated levels was associated with low cardiac performance in dengue patients in the different diseases phases.

Reviewer #2: Thanks to the authors for the modifications and answers to the questions, the manuscript reads much better with the changes.

We thank you for the time.
